# Study of Dangling Bond States in Magnetron-Sputtered a-Si Thin Films via Parametrization Using a Single UV–Vis–NIR Transmittance Spectrum

**DOI:** 10.3390/molecules31091469

**Published:** 2026-04-28

**Authors:** Dorian Minkov, George Angelov, Dimitar Nikolov, Rostislav Rusev, Eduardo Blanco, Susana Fernandez, Manuel Ballester, Emilio Marquez

**Affiliations:** 1 Scientific Research Section (NIS), Technical University, 1000 Sofia, Bulgaria; 2 Department of Microelectronics, Faculty of Electronics Engineering and Technologies, Technical University, 1000 Sofia, Bulgaria; angelov@ecad.tu-sofia.bg (G.A.);; 3 Department of Technology and Management of Communication Systems, Faculty of Telecommunications, Technical University, 1000 Sofia, Bulgaria; rusev@ecad.tu-sofia.bg; 4 Department of Condensed-Matter Physics, Faculty of Science, University of Cadiz, Puerto Real, 11510 Cadiz, Spain; eduardo.blanco@uca.es (E.B.); emilio.marquez@uca.es (E.M.); 5 Photovoltaic Solar Energy Unit, Centre for Energy, Environmental and Technological Research (CIEMAT), Avenida Complutense 40, 28040 Madrid, Spain; susanamaria.fernandez@ciemat.es; 6 Computer Science Department, Northwestern University, Evanston, IL 60208, USA; manuelballestermatito2021@u.northwestern.edu

**Keywords:** spectroscopy, parametrization of thin films, amorphous Si films, UV–Vis–NIR transmittance spectrum, electron transitions, dangling bonds, band tails, dielectric function, joint density of states (JDOS), density of states (DOS)

## Abstract

While both Urbach tails and dangling bonds are known to be present in a-Si films, the current literature lacks parametrization that simultaneously accounts for both types of defects using only transmittance spectra, reflectance spectra, or spectroscopic ellipsometry. To address this issue, we performed parametrizations of three magnetron-sputtered a-Si thin films deposited on glass substrates at different low pressures of argon gas, using only their measured UV–Vis–NIR transmittance spectra *T*(*λ* = [300, 2500] nm) and different dispersion models. We preprocessed *T*(*λ*) by suppressing both general and bandpass noise to yield the spectrum *T*_d_(*λ*). The films were parametrized from *T*_d_(*λ*) using two versions of the Tauc–Lorentz–Urbach dispersion model and the universal dispersion model (UDM) of Franta. The most accurate parametrization was achieved employing UDM including Urbach tail and three subgap oscillators. JDOS and the dielectric function *ε*(*E*) were computed by this UDM, and it was concluded that these three oscillators correspond to electron transitions via two bands of dangling bonds. The respective DOS is similar to the DOS previously reported for a-Si:H, but not to a-Si, indicating a relatively low density of dangling bonds in our a-Si films. Record low parametrization errors are achieved, which confirms the accuracy of these results.

## 1. Introduction

Most applications in optoelectronics, electro-optics, and optics use light from the UV–Vis–NIR spectral region and thin films [1,2,3]. This leads to the wide use of UV–Vis–NIR spectrophotometers and spectroscopic ellipsometers, which can measure transmittance spectrum or reflectance spectrum in this spectral region, for characterization of thin films [4,5,6,7].

In most cases, the measurement sample consists of the film formed on a non-transparent thick glass substrate of thickness d_s_ >> *λ*^2^/(2*n*_s_Δ*λ*), where *n*_s_(*λ*) is the refractive index of the substrate and Δ*λ* is the bandpass of the spectrophotometer or the ellipsometer [5,8]. In general, the thickness *d* of the film can vary over the light spot such that *d* ⊂ [$\bar{d}$ − ∆d, $\bar{d}$ + ∆d], where $\bar{d}$ is the average film thickness over the light spot and ∆d > 0 is the non-uniformity of the film [9]. The measured normal incidence UV–Vis–NIR transmittance spectrum of such a sample is denoted as *T*(*λ*); in spectrophotometry, it is commonly recorded from *λ*_max_ downward. The mathematical formula for the transmittance spectrum *T*(*λ*) of such a sample was developed by using the flow graphs approach [10], and it was shown that it is identical to the respective formula derived by the matrix approach [11].

In relation to the above, it is well known that the bandgap energies *E*_g_ of the vast majority of semiconductors and many dielectric materials correspond to photon energies within the UV–Vis–NIR spectral range [12,13]. This means that light from this range with photon energy *E* > *E*_g_ can be strongly absorbed in a semiconductor or dielectric film because it triggers transitions of electrons from the valence band into the conduction band [14]. Also, passing such light with *E* < *E*_g_ through such a film with average thickness *λ*/(2*n*) < $\bar{d}$ << *λ*^2^/(2*n*Δ*λ*), where *n*(*λ*) is the refractive index of the film and *E* (eV) ≈ 1239.8/*λ* (nm), causes thin-film interference, as revealed by the presence of interference maxima and minima in *T*(*λ*) [15,16]. Therefore, such a thin semiconductor or dielectric film is associated with the appearance of different geometrical features in its *T*(*λ*) and significant changes in its first derivative, which is favorable for accurate film characterization from *T*(*λ*).

In general, amorphous materials exhibit the Urbach tail, which is an exponential increase of the absorption coefficient *α* = 4π*k*/*λ* vs. *E* < *E*_g_, where *k* is the extinction coefficient, caused by structural disorder creating band tails near the conduction and valence band edges [17,18,19]. The degree of structural disorder in amorphous materials is proportional to the Urbach energy *E*_U_, which can be calculated from the slope of a linear section from the dependence log_10_(*α*) vs. *E* [20,21]. In this regard, a study of a thin a-Si:H film deposited by PECVD at 250 °C has reported *E*_g_ = 1.71 eV, *E*_U_ ~ 0.060 eV, a conduction band tail width of 0.37 eV, and a valence band tail width of 0.56 eV; thus, the valence band tail has been wider than the conduction band tail [22].

Additionally, according to the Mott–Davis model, tetrahedral amorphous materials with covalent bonding contain a significant concentration of dangling bonds, which are associated with both microvoids and vacancies [23]. Furthermore, a “neutral dangling bond” commonly exists in such an atom with one unpaired electron and a net neutral charge, while its electronic state is usually denoted by D0. Accordingly, a “positive dangling bond”, with electronic state D+, occurs after the unpaired electron has left its neutral dangling bond. Moreover, a “negative dangling bond”, with electronic state D−, arises when the neutral dangling bond has captured an extra electron, resulting in a net negative charge of the atom. Consequently, D+ can accept an electron, transitioning to D0, essentially acting as electron acceptor, while D− can donate an electron, transitioning to D0, acting as electron donor [24].

The electronic states corresponding to dangling bonds are localized states in the bandgap, whereby D+ is located above the Fermi level *E*_F_, while D− is below *E*_F_. Furthermore, a high density of D+ and D− pin *E*_F_ between them, according to the Defect Pool Model [25]. Partially overlapping bands of dangling bond states D+ and D− have been established in a-Si:H, where hydrogen atoms passivate some dangling bonds [26]. However, there are no publications about studying dangling bonds in non-hydrogenated amorphous silicon (a-Si) films by using only transmittance or reflectance spectrum.

The main practical applications of a-Si are in thin-film photovoltaic devices, thin-film transistor displays, anodes of lithium-ion batteries, and photocatalysts in hydrogen production from water [27,28]. Compared with a-Si:H with [3, 25]% hydrogen, a-Si has a higher concentration of dangling bonds, which makes its optical properties significantly dependent on the preparation method [29,30]. However, it was shown in [31] that a-Si thin films prepared by magnetron sputtering (MS) at Ar pressure of ≤1.1 Pa contain a negligible concentration of microvoids; therefore, they are expected to have not too high a concentration of dangling bonds [30]. This should be beneficial for practical applications because dangling bonds mostly act as non-radiative centers that trap charge and reduce mobility—unlike band tails, which, in general, are radiative centers [30]. In addition, obtaining a-Si films via magnetron sputtering is important because it provides higher film density and adhesion, as well as a low-temperature alternative, compared with those of thermal evaporation and plasma-enhanced chemical vapor deposition (PECVD) [28,31]. Notably, the analysis in [31] was performed assuming uniformity of the a-Si films, i.e., ∆d = 0, as well as transparency of the substrate, i.e., substrate extinction coefficient *k*_s_(*λ*) = 0.

The Tauc–Lorentz–Urbach dispersion model (TLU) has been employed for characterization of different amorphous semiconductor materials [32,33,34] since it includes the Urbach tail for *E* < *E*_g_, in addition to the Tauc–Lorentz model. In particular, MS a-Si thin films have been parametrized by the TLU dispersion model of Foldyna et al. (TLUF) [35,36], as well as by the TLU dispersion model of Rodriguez et al. (TLUR) [37,38]. The main difference between these two models is that the imaginary part of the dielectric function in the Urbach tail is expressed as *ε*_i_(*E*) = const/*E* × exp(*E*/*E*_U_) in TLUF [35] and *ε*_i_(*E*) = const × *E* × exp(*E*/*E*_U_) in TLUR [37]. However, both TLUF and TLUR are single oscillator models not including any oscillator with a central energy *E*_p_ < *E*_g_ (also known as a subgap oscillator), which means that they cannot describe accurately absorption involving dangling bonds. Unlike TLUF and TLUR, the universal dispersion model (UDM) of Franta et al. can include both Urbach tail and subgap Gaussian oscillators (GOs) [39,40], which can correspond to electron transitions via dangling bond states. One feature of UDM is that it considers electron transitions corresponding only to *E* > *E*_g_/2 as possibly due to the Urbach tail, unlike the assumption of *E* > 0 as possibly due to the Urbach tail in both TLUF and TLUR. Moreover, the UDM provides a formula for the joint density of states (JDOS), i.e., the number of pairs of electronic states—one occupied and one unoccupied—separated by photon energy *E*. Notably, all parameters included in TLUF, TLUR, and TLU should have values ≥ 0, according to [35,37,39].

The results from the optical characterization of MS a-Si films, from either *T*(*λ*) or reflectance spectrum *R*(*λ*), have been reported in [36,38,41,42]. Nevertheless, there is no published data from characterization of MS a-Si film by using a dispersion model (DM) that includes at least one subgap oscillator. However, a recent study using an advanced envelope method for *T*(*λ*) shows that a dispersion model employing only one oscillator with a central energy above *E*_g_ is insufficiently accurate for the subgap characterization of MS a-Si films [43].

Furthermore, every measured spectrum *T*(*λ*) contains some noise. To alleviate this problem, a method, abbreviated as SMEDM, was developed for superior suppression of general noise from *T*(*λ*) [44], utilizing a complete ensemble empirical mode decomposition with adaptive noise (CEEMDAN) [45]. In [45], SMEDM was applied to two model spectra *T*(*λ*), including four sets of model noises, and it was indicated that the relative error of the reconstructed noise did not exceed 26% for *T*(*λ*) > 0.01, with a bias of less than 22% from the effective noise level. Additionally, *T*(*λ*) includes a bandpass noise in the common case when the spectrophotometer or ellipsometer contains a monochromator [46]. Such monochromators typically have triangular bandpass functions, which allows for filtering the bandpass noise formulated by Equation (1) from [47]. Denoising of *T*(*λ*) for MS a-Si film samples was demonstrated in [43] by removing outliers from *T*(*λ*) using SMEDM and filtering the bandpass noise. In general, accurate characterization of a semiconductor or dielectric thin film from *T*(*λ*) in the UV–Vis–NIR region is difficult because, typically, *k*(*E* < *E*_g_) << *n*(*E* < *E*_g_), which hampers precise computation of *k*(*E* < *E*_g_). Based on the above, it is expected that denoising the *T*(*λ*) of such a film sample by employing only SMEDM and filtering bandpass noise should increase the accuracy of characterization of the film. However, such denoising has yet to be applied to thin-film parametrization via TLUF, TLUR, and the UDM.

In short, there are no published parametrizations of non-hydrogenated a-Si films derived solely by means of spectroscopic ellipsometry or spectrophotometry that take into account both Urbach tails and dangling bonds. The main goal of this work was to study subgap electron transitions (caused by absorption of photons with *E* < *E*_g_) in high-quality MS a-Si films prepared at low Ar pressure (≤1.1 Pa) using only the transmittance spectrum *T*(*λ*). A record low error in the parametrization of such films was achieved by employing the UDM including Urbach tail and three subgap GOs. The respectively computed JDOSs and the log_10_(α) vs. *E* plots reveal electron transitions via both Urbach tails and two bands of dangling bond states located close to the center of the bandgap.

## 2. Results

The same three MS a-Si thin film samples, A079, A031, and A072, and their respective measured spectra *T*(*λ*) used in [43] were employed in this study. To ensure consistency of the visualization, in all figures with three columns in this section, the panels in the first column refer to sample A079, those in the second to A031, and those in the third to A072. In addition, the vertical axis label is the same for all panels in a particular row in each one of these figures. Regarding the substrate, its transmissivity *x*_s_(*λ*) = exp(−4π*k*_s_(*λ*)d_s_/*λ*) represents the fraction of monochromatic light energy passing once between its two surfaces; therefore, *x*_s_(*λ*) ≥ *T*(*λ*).

### 2.1. Denoising of the Transmittance Spectra T(λ)

**Figure 1:**
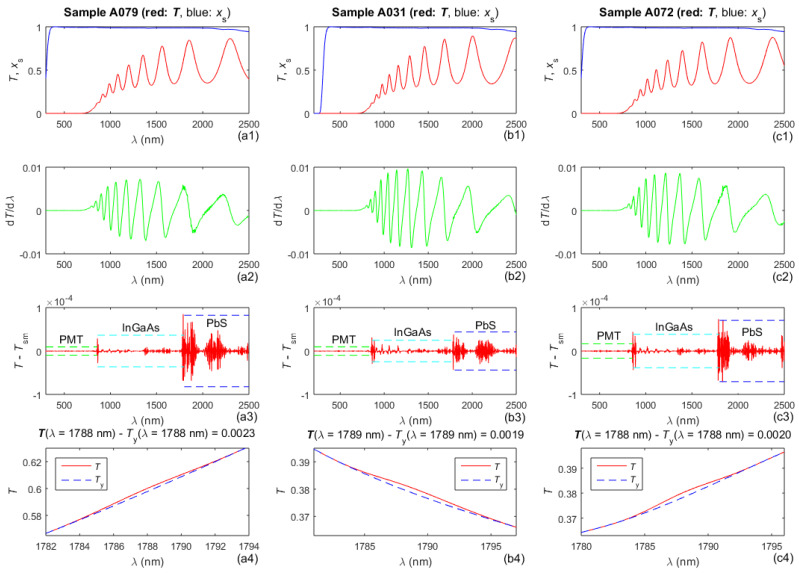
*T*(*λ*), *x*_s_(*λ*), and plots related to features of the general noise in *T*(*λ*).

*T*(*λ*), its first derivative d*T*/d*λ*, and *T*(*λ*) − *T*_sm_(*λ*) of the studied films are included in the first row, the second row, and the third row of Figure 1, respectively, where *T*_sm_(*λ*) was obtained by Savitzki–Golay smoothing of *T*(*λ*) [48]. Six sigma limits [49] of the difference *T*(*λ*) − *T*_sm_(*λ*) are depicted by differently colored dashed horizontal lines in the third row of Figure 1 over the spectral ranges covered by the employed detectors of PMT, InGaAs, and PbS, respectively. The fourth row of Figure 1 shows a magnified inset of *T*(*λ*) from the first row and of *T*_y_(*λ*), which is identical to *T*(*λ*) except for the visualized region just below 1800 nm, where it represents a smoothed *T*(*λ*) since │*T*(*λ*) − *T*_sm_(*λ*)│ has its largest value there.

Evidence of the general noise in *T*(*λ*) is seen by the apparent presence of noise in some regions of d*T*/d*λ* and *T*(*λ*) − *T*_sm_(*λ*). Furthermore, the curves for *x*_s_(*λ*) from the first row in Figure 1 show that there is noticeable absorption in the substrate for *λ* = [2000, 2500] nm, and the substrate is opaque for *λ* = [200, 250] nm. Therefore, it is expected that the consideration of substrate absorption in this study should result in improved accuracy of characterization of the a-Si film compared with [31], where the substrates were regarded as transparent. Additionally, the data from the third row in Figure 1 indicate that the general noise in *T*(*λ*) is the largest when the PbS detector is utilized and smallest when the PMT detector is used, as was already established in [43].

**Figure 2:**
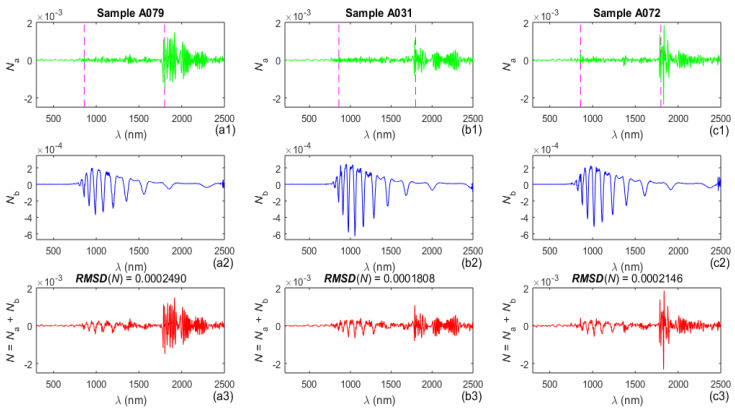
The noise in *T*(*λ*) and its components. General noise *N*_a_(*λ*) (**top row**), bandpass noise *N*_b_(*λ*) (**middle row**), and total noise *N*(*λ*) = *N*_a_(*λ*) + *N*_b_(*λ*) (**bottom row**). The vertical dashed pink lines in the top row correspond to replacements of the detectors.

In this study, outliers above the upper and below the lower dashed horizontal six sigma lines in the third row in Figure 1 were not removed from *T*(*λ*), unlike in [43], and the reasons for this are discussed later. The suppression of general noise from *T*(*λ*) was performed by using only SMEDM, as described in [44]. The general noise *N*_a_(*λ*) in *T*(*λ*) thus derived is shown in the top row of Figure 2. The bandpass noise *N*_b_(*λ*) was calculated using Equation (1) in [47]; applied to the measured transmittance spectrum with suppressed general noise, *T*(*λ*) − *N*_a_(*λ*); and is plotted in the middle row of Figure 2.

Based on the above, the denoised transmittance spectrum of a given sample is *T*_d_(*λ*) = *T*(*λ*) − *N*_a_(*λ*) − *N*_b_(*λ*). These denoised spectra *T*_d_(*λ*) were used thereafter, instead of *T*(*λ*), in the subsequent parametrizations of the three a-Si films. Similarly, each spectrum *T*_y_(*λ*) is denoised and its denoised spectrum is *T*_yd_(*λ*) = *T*_y_(*λ*) − *N*_y_(*λ*), where *N*_y_(*λ*) is the total noise in *T*_y_(*λ*). These spectra *T*_yd_(*λ*) were then used instead of *T*_y_(*λ*) in other parameterizations of the a-Si films.

### 2.2. Parametrization of the Three a-Si Films

Each of the TLUF, TLUR and UDM dispersion models provides formulae for the real part *ε*_r_(*E*) and the imaginary part *ε*_i_(*E*) of the dielectric function, using several parameters that depend on the dispersion model. These formulae for *ε*_r_(*E*) and *ε*_i_(*E*), as well as *n*_s_(*λ*), *k*_s_(*λ*), and d_s_, can be substituted into the expression for the transmittance spectrum of the sample from [10]. This allows for execution of the respective parametrization, which provides computed values of all the dispersion model parameters, the thickness parameters $\bar{d}$ and ∆d of the film, and the computed spectrum *T*_c_(*λ*) of the sample. In this paper, the following figure of merit

(1) $$FOM\left\{\left[\lambda (j_{2}),\lambda (j_{1})\right]\right\}=1000\times \sqrt{\frac{\sum_{j=j_{1}}^{j_{2}}{\left[T_{c}\left(\lambda (j)\right)-T_{d}\left(\lambda (j)\right)\right]}^{2}}{\left(j_{2}-j_{1}+1\right)}}\geq 0,$$

represents the parametrization error over the interval *λ* ⊂ [*λ*(*j*_2_), *λ*(*j*_1_)], where *j* is the number of the wavelength value counted sequentially from *λ*_max_. Therefore, a smaller *FOM* corresponds to a more accurate parametrization of a particular thin film, based on its *T*_d_(*λ*), over this interval.

The TLUF parametrizations were performed as described in [35]. The TLUR parametrizations are executed as in [37] using the damping parameter a = 0.0002. The computed parameters obtained from the TLUF and TLUR parametrizations of the films A079, A031, and A072 are listed in Table 1. The symbols representing these parameters in Table 1 are the same as those used in the papers [35,37] that present TLUF and TLUR, where *A* is the amplitude, *E*_0_ is the central energy, *C* is the broadening parameter, *E*_g_ is the optical bandgap, and *E*c is the boundary energy between the Tauc–Lorentz and Urbach regions.

The UDM parametrizations performed in this study use the dispersion model from [39], excluding high-energy valence electron excitations. This is because these excitations correspond to electron transitions with energies higher than the maximum photon energy for our measured spectra *T*(*λ*). Moreover, the electron transitions between the valence band and the conduction band are represented by a symmetrical term and one excitonic term, where the strength of these transitions is denoted by *N*_vc_. The parameters describing the symmetrical term are its relative strength *A*_0_ and the width of the conduction band *E*_h_ (*E*_h_ > *E*_g_), while those expressing the excitonic term are its relative strength *A*_1_, central energy *E*_c_ (*E*_c_ > *E*_g_), and broadening parameter *B*_c_ [39].

In this study, the following types of UDM parametrizations were performed: without GOs (abbreviated as UDM.0), with two subgap GOs (UDM.2), and with three subgap GOs (UDM.3). In the UDM, the parameters corresponding to the subgap electronic transitions are as follows: *N*_ut_ and *N*_pj_ are the strengths of the electron transitions via the Urbach tail states and the j-th GO; *E*_U_ and *E*_pj_ (*E*_pj_ < *E*_g_) are the Urbach energy and central energy of the j-th GO; and *B*_pj_ is the broadening parameter of the j-th GO.

The procedure for performing UDM.3 began with execution of UDM.0; its output parameters are then included in the initial approximation for execution of UDM.2. The output parameters from UDM.2 include parameters approximating the two strongest subgap GOs, and these output parameters were then included in the initial approximation for UDM.3. Thereafter, UDM.3 was executed three times with different initial approximations for the weakest subgap GO, which were selected so that its central energy is to the left of, to the right of, or between the central energies of the two strongest GOs obtained from UDM2. Out of these three executions, the output from UDM.3 was selected as the one yielding the smallest *FOM*. This procedure was designed to provide UDM.3 with a global minimum of the *FOM*. The parameters obtained by UDM.0 and UDM.3 parametrizations of the studied a-Si films are presented in Table 2, which also includes the sensitivity ranges corresponding to subgap electron transitions. For comparison, the following values have been obtained when employing the UDM, based on a multi-sample method combining ellipsometry and spectrophotometry, applied to a-Si:H films [40]: *N*_vc_ = 114.2, *E*_g_ = 1.82 eV, *A*_0_ = 1.0, *E*_h_ = 2.63 eV, *A*_1_ = 36, *E*_c_ = 3.50 eV, *N*_ut_ = 0.045, *E*_ut_ = 0.75 eV, *N*_p1_ = 1.7 × 10^−5^, *E*_p1_ = 1.39 eV, and *B*_p1_ = 0.134 eV.

**Figure 3:**
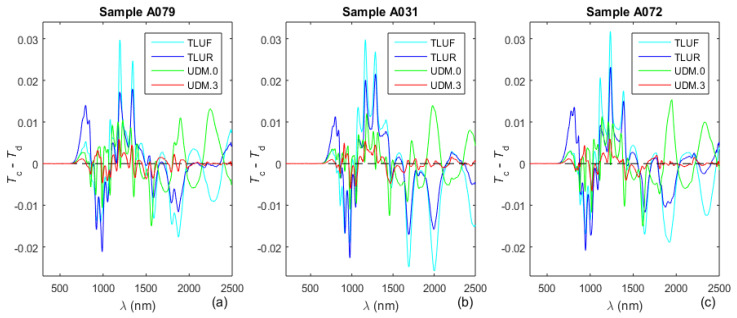
Difference between the computed transmittance spectrum *T*_c_(*λ*) and the denoised spectrum *T*_d_(*λ*) for the TLUF, TLUR, UDM.0, and UDM.3 parametrizations.

Since a more accurate parametrization using *T*_d_(*λ*) corresponds to a closer proximity of the computed spectrum *T*_c_(*λ*) to *T*_d_(*λ*) according to Equation (1), we evaluated this proximity directly. Therefore, the difference *T*_c_(*λ*) − *T*_d_(*λ*) is shown in Figure 3a–c for the a-Si film parametrizations featured in Table 1 and Table 2.

As seen from Table 1 and Table 2, the bandgap energy *E*_g_ was computed from each TLUF, TLUR, or UDM parametrization, which yielded a corresponding bandgap wavelength *λ*_g_ (nm) ≈ 1239.8/*E*_g_ (eV). Because the interference spectrum *T*(*λ*) profile differs significantly between the weak and strong absorption regions of the film, *FOM*s were calculated for *λ* > *λ*_g_, *λ* < *λ*_g_, and the entire range of *T*(*λ*). Such *FOM*s are included in Table 3 for all of the performed parametrizations of the three a-Si films.

Additionally, the quantities

(2) $$\begin{array}{l} D\left[FOM_{av}\left(A,B\right)\right]=\frac{\sum_{w=1}^{3}\left\{FOM\left[A\left(w\right)\right]-FOM\left[B\left(w\right)\right]\right\}}{3};\\ RD\left[FOM_{av}\left(A,B\right)\right]=\frac{D\left[FOM_{av}\left(A,B\right)\right]}{\sum_{w=1}^{3}\left\{FOM\left[A\left(w\right)\right]\right\}}\times 3\times 100(\%) \end{array}$$

were calculated using *FOM*s from the last column in Table 3, where A is one of the following parametrizations: TLUF, TLUR, UDM.0, UDM2, UDM.3, UDM.3y, or AEM; B is a parametrization providing *FOM*[B(w)] < *FOM*[A(w)]; and w = 1, 2, or 3 corresponds to each of the three a-Si films. D[*FOM*_av_(A,B)] is the difference between the *FOM*s from the parametrization A and the parametrization B, averaged over these a-Si films, while RD[*FOM*_av_(A,B)] is the relative difference representing D[*FOM*_av_(A,B)] averaged over these films. D[*FOM*_av_(A,B)] and RD[*FOM*_av_(A,B)] calculated from Equation (2), for different parametrizations A and B, are presented in Table 4.

**Figure 4:**
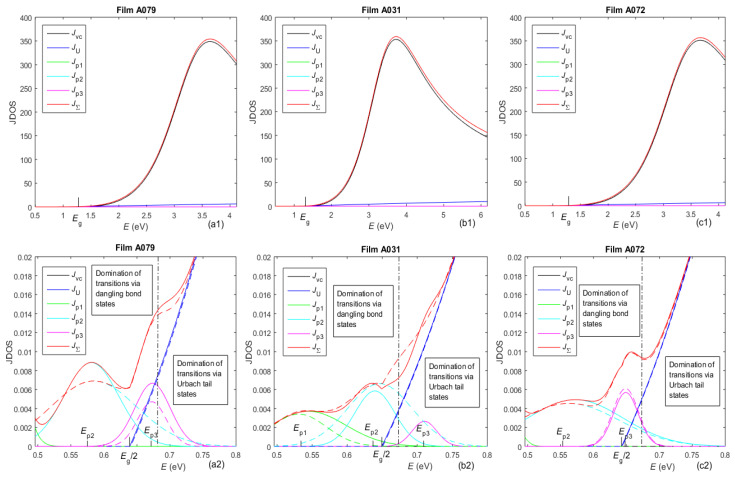
JDOSs for the different types of electron transitions in UDM.3 and UDM.2. The panels in the bottom row represent magnified images of those in the top row, but for *E* < *E*_g_. The solid lines, as well as the marks for *E*_g_, *E*_g_/2, and *E*_pj_ correspond to UDM.3, and the correspondingly colored dashed lines to UDM.2. Band-to-band transitions dominate the spectral region *E* > *E*_g_, and the boundary between the spectral regions dominated by transitions via Urbach tail states and via dangling bond states is illustrated by the vertical dash–dot lines in the panels from the bottom row.

The following types of electron transitions were considered, e.g., in UDM.3: from the valence band to the conduction band, via Urbach tails, and via each one of the three GOs, according to [39]. The computed JDOSs are designated as *J*_vc_, *J*_U_, *J*_p1_, *J*_p2_, and *J*_p3_. Their sum *J*_Σ_ represents the total JDOS, and all of these values are shown in Figure 4. The boundary between the spectral regions dominated by electron transitions via Urbach tail states and those via dangling bonds was determined from the equation *J*_U_ = *J*_p1_ + *J*_p2_ + *J*_p3_.

**Figure 5:**
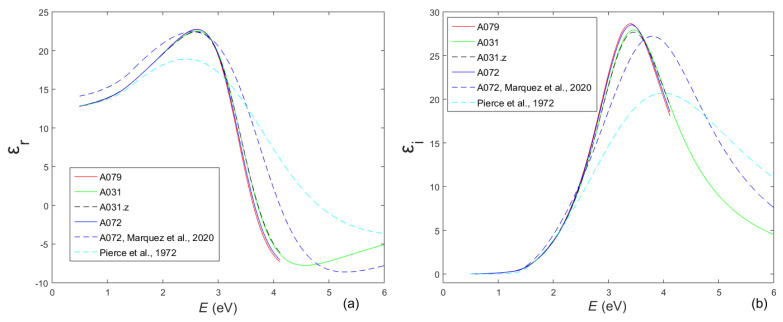
Computed (**a**) real part *ε*_r_(*E*) and (**b**) imaginary part *ε*_i_(*E*) of the dielectric function. Data for the three a-Si films were obtained using UDM.3 parametrizations; specifically, the UDM.3z parametrization for film A031 is denoted here as A031.z. Results for the film A072, obtained via spectroscopic ellipsometry by Marquez et al. [41], and for the electron-gun-evaporated a-Si from Pierce et al. [50] are also included.

The real part *ε*_r_ and the imaginary part *ε*_i_ of the dielectric function of the studied a-Si films, computed by UDM.3, are included in Figure 5.

**Figure 6:**
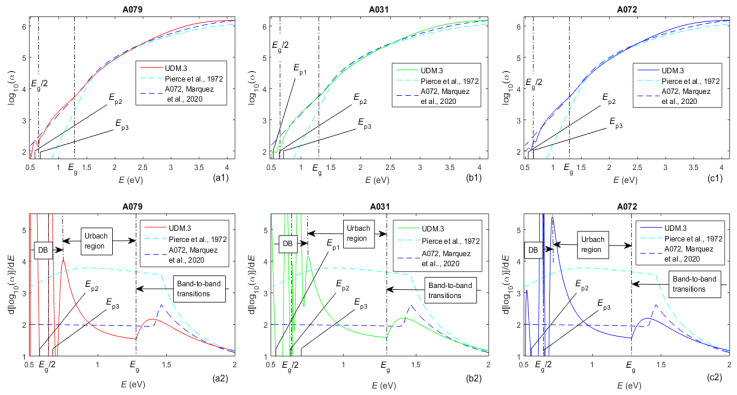
Two kinds of graphs related to *α*(*E*) computed via UDM.3 for the a-Si films. The respective *E*_g_, *E*_g_/2, and *E*_pj_, as well as the types of electron transitions, are also shown, where the labels DB in the second-row panels refer to the spectral region where the transitions via dangling bond states are prominent. Results for the film A072, obtained via spectroscopic ellipsometry by Marquez et al. [41], and for the electron-gun-evaporated a-Si from Pierce et al. [50] are also included.

Graphs associated with the absorption coefficient *α*(*E*) of the three a-Si films, calculated from *k*(*E*) determined using UDM.3, are shown in Figure 6.

## 3. Discussion

In general, *T*_d_(*λ*_min_ < *λ* < *λ*_g_) is significantly smaller than *T*_d_(*λ* > *λ*_g_) for interference transmittance spectra *T*(*λ*) [15,31], e.g., as derived from *E*_g_ in Table 1 and Table 2 and seen from the graphs in the top row in Figure 1. This indicates that such interference spectra *T*(*λ*) are particularly suitable for accurate subgap parametrization because the relative error of *T*_c_(*λ* > *λ*_g_) should be smaller than that of *T*_c_(*λ*_min_ < *λ* < *λ*_g_) to provide a small *FOM*, according to Equation (1).

The plots in Figure 3a–c show that the fit of the computed transmittance spectrum *T*_c_(*λ*) to *T*_d_(*λ*) improves according to the following order of parametrizations for all studied a-Si films: TLUF, TLUR, UDM.0, and UDM.3. Therefore, the accuracy of parametrization of these films should increase in the same order; thus, TLUF should provide the least accurate parametrization, and UDM.3 should be the most accurate amongst these parametrizations. Notably, it is seen from Table 1 and Table 2 that all parameters computed using TLUF, TLUR, or the UDM have values ≥ 0, which is a requirement of these dispersion models, thus confirming the validity of the computed parameters.

The smaller values of *FOM* for TLUR compared with those for TLUF, from the last column in Table 3, reconfirm that TLUR provides more accurate parametrization than TLUF for all studied a-Si films, which is consistent with the results from [38]. It was also concluded from there that TLUR parametrization of a given a-Si film is less accurate than each UDM parametrization of that film. In particular, *FOM* for the UDM.0 parametrization, i.e., without GOs, is smaller than that for TLUF or TLUR, for each of the a-Si films. This indicates that the consideration of Urbach tail transitions only for *E* > *E*_g_/2, as in UDM [39], is more accurate than assuming Urbach tail transitions, even for smaller energies *E* > 0, as in TLUF and TLUR [35,37], in the studied a-Si films.

It is also seen from the last column in Table 3 that *FOM*(UDM.0) > *FOM*(UDM.2) > *FOM*(UDM.3) for each of the a-Si films. This shows that including three GOs in the UDM results in more accurate parametrization of these films than the UDM without such oscillators and the UDM with two GOs. Also, the data about RD[*FOM*_av_(A,B)] from Table 4 indicate that the most significant improvement in the accuracy of parametrization amongst the parametrizations featured in Table 3 is achieved by adding two GOs to UDM.0. Furthermore, the addition of a third GO also improves the accuracy but significantly less than the above since RD[*FOM*_av_(UDM.2,UDM.3)] = 0.44 << RD[*FOM*_av_(UDM.0,UDM.2] = 7.69. Moreover, RD[*FOM*_av_(AEM,UDM.3)] = 4.85, implying that the parametrizations by UDM with 3 GOs provide significantly more accurate data for the three a-Si films compared with their previous most accurate characterization by AEM from [43]. The discussion in this paragraph indicates that parametrization of the three a-Si films from *T*_d_(*λ*) using the UDM with three GOs provides the most accurate characterization of MS a-Si films published so far.

According to the panels in the bottom row in Figure 1, max{│*T*(*λ*) − *T*_y_(*λ*)│} equals 0.0023 for the sample A079, 0.0019 for A031, and 0.0020 for A072. After the suppression of general noise by SMEDM, the value corresponding to the above function decreases by factors of 3.22, 2.79, and 4.05 for A079, A031, and A072, respectively. In addition, the computed film thickness parameters $\bar{d}$ and ∆d essentially do not depend on whether they are obtained by employing UDM.3 or UDM.3y, as seen from Table 3. Moreover, the RD[*FOM*_av_(UDM3,UDM.3y)] = 0.04 from Table 3 is very small. The above results indicate that the suppression of general noise by SMEDM also suppresses outliers in *T*(*λ*) for these a-Si samples, thus justifying why outliers are not removed from *T*(*λ*) in this study, unlike in [43].

In addition, the main source of systematic instrumental error in the measurement of *T*(*λ*) is the photometric non-linearity, which is Δ*T*(*λ* > 1000 nm, T = 0.8) = ± 0.00055 and Δ*T*(*λ* > 1000 nm, T = 0.4) = ±0.00028 for our spectrophotometer, the Perkin-Elmer Lambda 1050 UV/visible/NIR [51]. In fact, this error is reduced further by the use of SMEDM in this study, as discussed in the last paragraph. With regard to this, we evaluated a computed spectrum *T*_c0_(*λ*) by using the parameters obtained by UDM.3, as presented in Table 2, excluding all three GOs for each of the three a-Si films. It turns out that the difference *T*_c0_(*λ*) − *T*_c_(*λ*) reaches the largest values [0.025, 0.03] for *λ* > 2*λ*_g_ around the maxima of *T*_c_(*λ*) for all three films. These data show that the contribution of the three GOs to *T*(*λ*) is much bigger than that of the photometric non-linearity and reconfirm the validity of the computed results in this study.

Data from Table 2 also show that the strengths of the electron transitions, computed by UDM.3, have the following orders of magnitude: *N*_vc_ ~ 320, *N*_ut_ ~ 20, and *N*_pj_ = [1 × 10^−4^, 3 × 10^−3^]. Such strong electron transitions from the valence to conduction band, medium strength transitions via Urbach tail states, and weak transitions via dangling bond states can be expected for a-Si:H [25,26] and perhaps for a-Si with a small concentration of microvoids [31]. This assertion is confirmed by the fact that the central energies *E*_pj_ of all GOs, computed by UDM.3, are smaller than *E*_g_, as seen from Table 2 and Figure 4. Moreover, the data from Table 2 and Figure 4 show that the central energy *E*_p1_ is significantly smaller than *E*_g_/2, *E*_p2_ is slightly smaller than *E*_g_/2, and *E*_p3_ is slightly larger than *E*_g_/2.

The above results about the strengths *N*_pj_ and the central energies *E*_pj_ of the three GOs show that the GOs with central energies *E*_p2_ and *E*_p3_ correspond to electron transitions via two dangling bond bands, and the GO with a central energy *E*_p1_ is attributed to electron transitions between these two dangling bond bands. In addition, the ratio of the strengths of the electron transitions via dangling bond states to those via tail states is *N*_p_/*N*_ut_ ~ 20 × 10^−4^/20 = 1 × 10^−4^, as seen from Table 2, which shows that the concentration of the dangling bonds is much smaller than that of the band tails.

**Figure 7:**
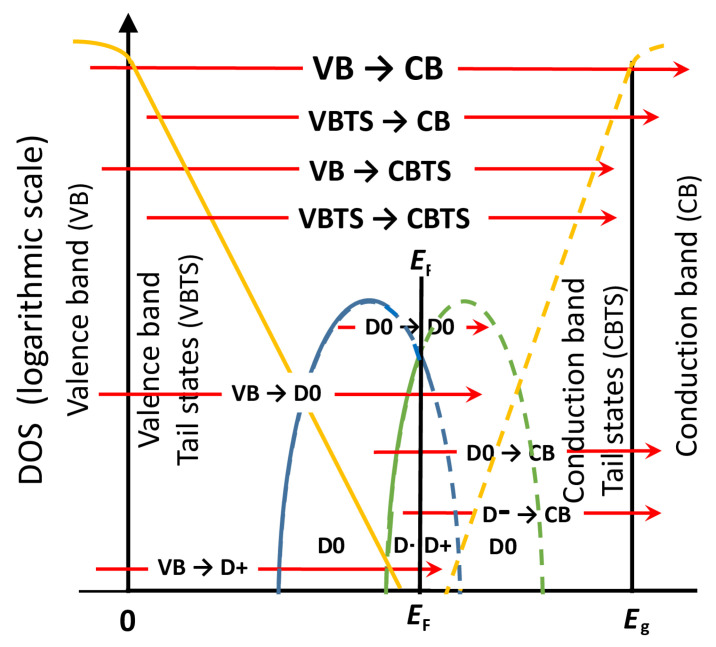
Logarithmic graph of the DOS energy spectrum and electron transitions upon illumination in the studied MS a-Si thin films. The area enclosed by the blue line represents the band of dangling bond states D+ and D0, while the area enclosed by the green line depicts the band of dangling bond states D− and other D0. The solid and dashed yellow lines indicate the valence band tail states and the conduction band tail states, respectively. The electron transitions upon illumination are depicted by red arrows. Electronic states below *E*_F_ are represented by solid lines because they can emit electrons, whereas states above *E*_F_ are shown by dashed lines since they can absorb electrons.

Taking into account that a-Si has a higher concentration of dangling bonds than a-Si:H with [3, 25]% hydrogen [29,30], it can be inferred by analogy with well-established literature about the application of the Defect Pool Model to a-Si:H [25] that the Fermi level *E*_F_ should be located between these two dangling bond bands in the absence of illumination. In addition, it can be presumed that the valence band tail is wider than the conduction band tail, by analogy with the already discussed data about a-Si:H from [22]. These results are consistent with the DOS and electron transitions upon illumination, as illustrated in Figure 7.

Additionally, the relative length of a particular red arrow in Figure 7 identifies which of the three GOs employed by UDM.3 correspond to the respective electronic transition. Therefore, the electron transitions D0 → D0 are described by the GO with central energy *E*_p1_. Moreover, the transitions D0 → CB and D- → CB are represented by the oscillator with central energy *E*_p2_, and the transitions VB → D0 and VB → D+ are represented by the oscillator with central energy *E*_p3_. Notably, this kind of DOS energy spectrum and electron transitions involving dangling bonds have been applied to a-Si:H [22,52], but they have not been used for a-Si.

The static refractive index *n*_v_ = *n*(*E* → 0) of the studied film is determined by using *n*(*λ*), as obtained by UDM.3, and the Wemple–DiDomenico plot depicting (*n*^2^ − 1)^−1^ vs. *E*^2^, similarly to that in [31]. Furthermore, the volume fraction of voids *f*_void_ is calculated by means of the Bruggeman effective medium approximation, employing *n*_v_ and the static refractive index *n*_0_ = 3.697 of pure a-Si without voids (derived from data in [53]), as described in [31]. Such calculations provide *f*_void_ = 6.7% for the film A079, *f*_void_ = 6.1% for A031, and *f*_void_ = 6.4% for A072. These low values of *f*_void_ should lead to a relatively low concentration of dangling bonds and a higher material density compared with a-Si films with a larger *f*_void_. Moreover, the higher material density of the films studied here should result in a higher refractive index *n* according to the Lorentz–Lorentz relation [54], as well as a relatively high real part *ε*_r_ = *n*^2^ − *k*^2^ and imaginary part *ε*_2_ = 2*nk* of their dielectric function. Therefore, the relatively low peaks of *ε*_r_ and *ε*_i_ from [50] in Figure 5 are attributed to a lower material density of electron-gun-evaporated a-Si compared with MS a-Si, which is in accordance with the results from [55].

On the other hand, *ε*_i_(*E* < *E*_g_) for A072, obtained in [41] by spectroscopic ellipsometry and assuming validity of the Cody–Lorentz dispersion model, is significantly higher than our corresponding result for the same film, as seen from Figure 5a. However, MS a-Si films were characterized in [41], and for each one, the computed average film thickness $\bar{d}$ was smaller than the value measured by SEM. This indicates that the refractive index *n*(*E* < *E*_g_) and *ε*_r_(*E* < *E*_g_) computed in [41] are larger than their respective true values, according to the interference fringes equation, 2$\bar{d}$*n*(*E*_t_ < *E*_g_) = const(*E*_t_) [16], where *E*_t_ corresponds to a tangent point between *T*_d_(*λ*) and its two envelopes. This discrepancy of results from [41] is caused by an apparently insufficient accuracy of the Cody–Lorentz dispersion model for MS a-Si films.

Furthermore, the results computed by UDM.3 and presented in Figure 5 show that the dielectric functions of the three MS a-Si films are very similar over the interval *λ* = [300, 2500] nm, although these films were prepared at different Ar gas pressures. In addition, the curves denoted by A031 (computed for *λ* = [200, 2500] nm) and A031.z (computed for *λ* = [300, 2500] nm) in Figure 5 are almost identical over the interval *λ* = [300, 2500] nm. This result and data from Table 3 indicate that the characteristics of the film A031 computed by UDM.3 over the interval *λ* = [300, 2500] nm are practically independent of whether the parametrization is executed over this interval or over the wider interval *λ* = [200, 2500] nm.

Notably, the TLUF, TLUR and Cody–Lorentz dispersion models assume the existence of a Urbach tail for *E* < *E*_g_, which makes them applicable to amorphous materials [56]. Therefore, utilization of these dispersion models results in a linear relationship between log_10_(α) and *E* for *E* < *E*_g_ [56]. This is visualized by the blue dashed lines in the top row of Figure 6, which represent the Cody–Lorentz model characterization of film A072 from [41]. However, the curves corresponding to UDM.3 characterizations of the three a-Si films, also from the top row of Figure 6, indicate that the absorption is actually weaker for *E* < *E*_g_/2, which is attributed to electron transitions via the dangling bond bands.

According to the Tauc equation, (α*E*)^1/*q*^ = const × (*E* − *E*_g_), where *q* = 1/2 for direct allowed electron transitions, *q* = 3/2 for direct forbidden transitions, *q* = 2 for indirect allowed transitions, and *q* = 3 for indirect forbidden transitions [57]. On the other hand, the first-order Taylor series expansion of the Tauc equation provides *q* ≈ d[log_10_(*αE*)]/d*E* [58]; correspondingly, this function drawn in the bottom row of Figure 6 represents an approximation of *q*. Therefore, it was concluded, from the solid curves in the bottom row of Figure 6, that the dominant electron transitions with *E* close above *E*_g_ are with *q* ≈ 2, i.e., indirect allowed transitions from the top of the valence band to the bottom of the conduction band, as in crystalline Si [55]. Additionally, the solid lines from the bottom row of Figure 6 are heavily folded for *E* = [0.5, 0.7] eV, with maxima above 6 and minima below 0, thus confirming involvement of dangling bond states in the electron transitions. It is also seen from there that the dominant electron transitions are indirectly forbidden for *E* = [0.75, 0.85] eV and directly forbidden for *E* = [1.1, *E*_g_] eV, as both should involve band tail states. Furthermore, the dominant electron transitions are also direct forbidden for *E* = [1.7, 1.9] eV, with contributions from direct allowed transitions above this energy range. It is recognized that the dangling bond bands in MS a-Si thin films, as discussed in this section, are identified solely through optical data from *T*(*λ*) and modeling assumptions, without independent defect-sensitive measurements.

In short, our results provide the dielectric function in the UV–Vis–NIR spectral region of a-Si thin films, taking into account both tail states and dangling bond states, as well as the volume fraction of voids, by using only the spectrum *T*(*λ*) of the sample. On the other hand, in a-Si:H, hydrogen atoms attach to dangling bonds, which leads to large decrease in both the structural disorder and the concentration of dangling bonds (by several orders) [22]. Indeed, *E*_U_ ~ 220 meV in this study of a-Si and *E*_U_ ~ 60 meV for a-Si:H in [22]. Therefore, some studies of a-Si:H thin films use dielectric functions corresponding to a combination of Tauc–Lorentz oscillators at energies above *E*_g_, while disregarding Urbach tails and dangling bonds, but including voids to represent surface layers [59,60,61].

Furthermore, photocatalytic films typically have high porosity to yield maximum active sites for the reaction and relatively wide bandgaps providing strong chemical stability and high redox potential [62]. In this respect, the *E*_g_ of photocatalytic films have been determined by using both UV–Vis and diffuse reflectance spectroscopy, while disregarding tail states and dangling bond states, whose spectroscopic features are mostly in the NIR spectral region [63]. However, studying such subgap states similar to the one in this paper would be beneficial for broadening the light absorption and controlling charge carrier lifetime, which is a bottleneck in photocatalytic efficiency [62].

## 4. Materials and Methods

Each of the samples was prepared by magnetron sputtering with Ar gas to produce an a-Si thin film on Corning Glass Eagle XG substrate with thickness d*_s_* = 1 mm. Three such samples were used, whereby Ar gas pressure of *p*_Ar_ = 0.1 Pa was supplied for the film from sample A079, *p*_Ar_ = 0.7 Pa for the film from sample A031, and *p*_Ar_ = 1.1 Pa for the film from sample A072. All the depositions were performed at room temperature, and the value of the RF power applied was 525 W.

The transmittance spectrum *T*(*λ*) of each sample was measured using a Perkin-Elmer Lambda 1050 UV/visible/NIR double-beam spectrophotometer (Waltham, MA, USA) covering the spectral range 175–3300 nm. The measurements were performed at normal incidence to the film with an illuminated film area of 10 mm × 3 mm. The spectral range was λ = [200, 2500] nm for the measurements of the samples A072 and A079, and λ = [300, 2500] nm for the sample A031. Notably, accurate measurement of T(λ < 200 nm) would require a high-grade nitrogen purge or a vacuum system, and the substrate absorbs much more strongly for λ > 2500 nm. The measurements of each T(λ) were conducted with the following detectors as the wavelength λ was decreasing: a PbS detector for the range [1801, 2500] nm, an InGaAs detector for [861, 1800] nm, and a PMT for [min(λ), 860] nm. The wavelength step is 1 nm in this study. Further details about the preparation of the samples and the measurement of their transmittance spectra *T*(*λ*) have been reported in [31].

The normal incidence transmittance spectrum *T_s_*(*λ*) and quasi normal incidence reflectance spectrum *R_s_*(*λ*) of a 1 mm thick bare Corning Glass Eagle XG substrate were also measured. The refractive index *n*_s_(*λ*) and the extinction coefficient *k*_s_(*λ*) of the substrate are computed by solving a system of two equations, each of them equating *T_s_*(*λ*) or *R_s_*(*λ*) with its respective formula from Equation (3) or Equation (4) in [11].

## 5. Conclusions

The most accurate parametrizations of the studied MS a-Si thin films were achieved by employing the dispersion model UDM.3, including an Urbach tail and three subgap oscillators. Analysis of the respective JDOS indicated that they are associated with electron transitions via both tail states and dangling bonds states, as shown in Figure 7. Notably, this distinction between electron transitions via dangling bonds and band tails, alongside the analysis of thickness and dielectric function, offers a new perspective on amorphous films, derived solely from a single transmittance or reflectance spectrum.

The approach and results described in this paper can be used in the design of devices involving a-Si films, as dangling bonds commonly lead to non-radiative recombination and decreased mobility, unlike tail states. In the future, such studies can be performed for thin amorphous films with different compositions.

## Figures and Tables

**Table 1:** Computed parameters derived from the TLUF and TLUR parametrizations of the three studied a-Si films.

**DM**	**Film A079**						
TLUF	*A* (eV)	*E*_0_ (eV)	*C* (eV)	*E*_g_ (eV)	*E*_c_ (eV)	$\bar{d}$ (nm)	Δd (nm)
	101.7	3.442	1.628	1.064	1.585	1305.6	14.8
TLUR	*A* (eV)	*E*_0_ (eV)	*C* (eV)	*E*_g_ (eV)	*E*_c_ (eV)	$\bar{d}$ (nm)	Δd (nm)
	102.7	3.413	1.434	1.069	1.682	1286.7	20.0
**DM**	**Film A031**						
TLUF	*A* (eV)	*E*_0_ (eV)	*C* (eV)	*E*_g_ (eV)	*E*_c_ (eV)	$\bar{d}$ (nm)	Δd (nm)
	96.67	3.488	1.434	1.009	1.491	1400.2	0
TLUR	*A* (eV)	*E*_0_ (eV)	*C* (eV)	*E*_g_ (eV)	*E*_c_ (eV)	$\bar{d}$ (nm)	Δd (nm)
	104.7	3.463	1.505	1.076	1.654	1395.5	0
**DM**	**Film A072**						
TLUF	*A* (eV)	*E*_0_ (eV)	*C* (eV)	*E*_g_ (eV)	*E*_c_ (eV)	$\bar{d}$ (nm)	Δd (nm)
	100.1	3.457	1.566	1.047	1.548	1346.9	0
TLUR	*A* (eV)	*E*_0_ (eV)	*C* (eV)	*E*_g_ (eV)	*E*_c_ (eV)	$\bar{d}$ (nm)	Δd (nm)
	100.6	3.413	1.389	1.009	1.491	1338.3	6.2

**Table 2:** Computed parameters from the UDM.0 and UDM.3 parametrizations of the a-Si films.

**DM**	**Film A079**						
UDM.0	*N*_vc_	*A*_0_	*A*_1_	*E*_g_ (eV)	*E*_c_ (eV)	*B*_c_ (eV)	*E*_h_ (eV)
	328.6	0.8952	22.10	1.266	3.320	0.8256	55.62
	*N*_ut_	*E*_U_ (eV)	$\bar{d}$ (nm)	Δd (nm)			
	27.51	0.2181	1249.9	26.9			
UDM.3	*N*_vc_	*A*_0_	*A*_1_	*E*_g_ (eV)	*E*_c_ (eV)	*B*_c_ (eV)	*E*_h_ (eV)
	314.6	0.9644	11.87	1.278 ± 0.025%	3.285	0.8735	44.69
	*N*_ut_	*E*_U_ (eV)	*N*_p1_	*E*_p1_ (eV)	*B*_p1_ (eV)	*N*_p2_	*E*_p2_ (eV)
	20.78 ± 0.49%	0.2394 ± 0.30%	2.633 × 10^−3^ ± 9.1%	0.4460 ± 0.41%	0.02187 ± 6.0%	1.577 × 10^−3^ ± 4.5%	0.5751 ± 1.0%
	*B*_p2_ (eV)	*N*_p3_	*E*_p3_ (eV)	*B*_p3_ (eV)	$\bar{d}$ (nm)	Δd (nm)	
	0.04134 ± 4.6%	6.680 × 10^−4^ ± 11%	0.6714 ± 0.96%	0.02697 ± 2.4%	1281.1 ± 0.042%	20.8 ± 4.9%	
**DM**	**Film A031**						
UDM.0	*N*_vc_	*A*_0_	*A*_1_	*E*_g_ (eV)	*E*_c_ (eV)	*B*_c_ (eV)	*E*_h_ (eV)
	250.9	−0.0002	0.01467	1.298	3.653	0.7225	10.46
	*N*_ut_	*E*_U_ (eV)	$\bar{d}$ (nm)	Δd (nm)			
	3.773	0.2184	1363.8	14.8			
UDM.3	*N*_vc_	*A*_0_	*A*_1_	*E*_g_ (eV)	*E*_c_ (eV)	*B*_c_ (eV)	*E*_h_ (eV)
	320.0	0.6883	17.75	1.299 ± 0.013%	3.326	0.9326	44.90
	*N*_ut_	*E*_U_ (eV)	*N*_p1_	*E*_p1_ (eV)	*B*_p1_ (eV)	*N*_p2_	*E*_p2_ (eV)
	22.09 ± 0.23%	0.2325 ± 0.21%	9.144 × 10^−4^ ± 10%	0.5342 ± 3.8%	0.05361 ± 11%	6.527 × 10^−4^ ± 1.6%	0.6369 ± 0.60%
	*B*_p2_ (eV)	*N*_p3_	*E*_p3_ (eV)	*B*_p3_ (eV)	$\bar{d}$ (nm)	Δd (nm)	
	0.02848 ± 3.9%	1.658 × 10^−4^ ± 12%	0.7093 ± 1.0%	0.01774 ± 12%	1388.0 ± 0.012%	0	
**DM**	**Film A072**						
UDM.0	*N*_vc_	*A*_0_	*A*_1_	*E*_g_ (eV)	*E*_c_ (eV)	*B*_c_ (eV)	*E*_h_ (eV)
	269.1	−1.839	31.46	1.270	3.421	0.8092	24.83
	*N*_ut_	*E*_U_ (eV)	$\bar{d}$ (nm)	Δd (nm)			
	10.48	0.2164	1299.4	19.7			
UDM.3	*N*_vc_	*A*_0_	*A*_1_	*E*_g_ (eV)	*E*_c_ (eV)	*B*_c_ (eV)	*E*_h_ (eV)
	312.4	0.9181	19.14	1.285 ± 0.51%	3.298	0.8909	45.44
	*N*_ut_	*E*_U_ (eV)	*N*_p1_	*E*_p1_ (eV)	*B*_p1_ (eV)	*N*_p2_	*E*_p2_ (eV)
	21.51 ± 0.25%	0.2346 ± 0.083%	1.093 × 10^−4^ ± 11%	0.4821 ± 0.074%	0.01255 ± 12%	1.579 × 10^−3^ ± 7.4%	0.5544 ± 3.7%
	*B*_p2_ (eV)	*N*_p3_	*E*_p3_ (eV)	*B*_p3_ (eV)	$\bar{d}$ (nm)	Δd (nm)	
	0.07308 ± 7.6%	4.123 × 10^−4^ ± 2.4%	0.6479 ± 11.0%	0.01895 ± 2.5%	1325.8 ± 0.068%	11.2 ± 3.6%	

**Table 3:** Computed film parameters and *FOM*s calculated from Equation (1). The dispersion model UDM.3y utilized the denoised spectrum *T*_yd_(*λ*) and three GOs. UDM.3z employed the denoised spectrum *T*_d_(*λ*) of the sample A031, though only over the narrower interval *λ* = [300, 2500] nm, and three GOs. AEM * refers to the advanced envelope method from [43].

	**Film A079**					
**Dispersion Model**	**min(** ***λ*** **)**	$\bar{d}$ ** (nm), Δd (nm)**	***λ*** **_g_** ** (nm)**	***FOM*** ** (** ***λ*** ** < *λ*** **_g_** **)**	***FOM*** ** (** ***λ*** ** > *λ*** **_g_** **)**	***FOM*** ** (all** *** λ*** **)**
TLUF	300	1305.6, 14.8	1165	5.056	8.741	7.531
TLUR	300	1286.7, 20.0	1160	6.725	5.727	6.138
AEM *	665	1282.6, 20.0	1008	0.513	3.594	3.296
UDM.0	300	1249.9, 26.9	980	1.850	6.094	5.169
UDM.2	300	1277.9, 21.1	968	0.925	1.777	1.568
UDM.3	300	1281.1, 20.8	970	0.916	1.627	1.448
UDM.3y	300	1281.0, 20.8	970	0.922	1.618	1.442
	**Film A031**					
**Dispersion Model**	**min(** ***λ*** **)**	$\bar{d}$ ** (nm), Δd (nm)**	***λ*** **_g_** ** (nm)**	***FOM*** ** (** ***λ*** ** < *λ*** **_g_** **)**	***FOM*** ** (** ***λ*** ** > *λ*** **_g_** **)**	***FOM*** ** (all** *** λ*** **)**
TLUF	200	1400.2, 0	1228	7.884	10.96	9.708
TLUR	200	1395.5, 0	1152	5.936	7.869	7.129
AEM *	683	1382.9, 0	1001	0.866	2.119	1.959
UDM.0	200	1363.8, 14.8	955	1.438	5.701	4.745
UDM.2	200	1389.0, 0	953	1.008	1.903	1.664
UDM.3	200	1388.0, 0	955	0.913	1.869	1.618
UDM.3y	200	1388.1, 0	954	0.984	1.845	1.615
UDM.3z	300	1388.0, 0	953	1.039	1.848	1.650
	**Film A072**					
**Dispersion Model**	**min(** ***λ*** **)**	$\bar{d}$ ** (nm), Δd (nm)**	***λ*** **_g_** ** (nm)**	***FOM*** ** (** ***λ*** ** < *λ*** **_g_** **)**	***FOM*** ** (** ***λ*** ** > *λ*** **_g_** **)**	***FOM*** ** (all** *** λ*** **)**
TLUF	300	1346.9, 0	1184	6.057	9.603	8.362
TLUR	300	1338.3, 6.2	1167	6.959	6.100	6.453
AEM *	699	1329.5, 8.6	1000	0.798	2.910	2.675
UDM.0	300	1299.4, 19.7	976	1.772	6.156	5.218
UDM.2	300	1324.7, 11.3	964	0.923	1.590	1.422
UDM.3	300	1325.9, 11.0	965	0.914	1.569	1.404
UDM.3y	300	1325.9, 11.0	965	0.917	1.578	1.412

**Table 4:** Values of D[*FOM*_av_(A,B)] and RD[*FOM*_av_(A,B)] for different parametrizations A and B.

D[*FOM*_av_(A,B)] [TLUF,TLUR]	D[*FOM*_av_(A,B)] [TLUR,UDM.0]	D[*FOM*_av_(A,B)] [UDM.0,UDM.2]	D[*FOM*_av_(A,B)] [UDM.2,UDM.3]	D[*FOM*_av_(A,B)] [UDM.3,UDM.3y]	D[*FOM*_av_(A,B)] [AEM,UDM.3]
1.960	1.527	3.493	0.061	0.006	1.153
RD[*FOM*_av_(A,B)] [TLUF,TLUR]	RD[*FOM*_av_(A,B)] [TLUR,UDM.0]	RD[*FOM*_av_(A,B)] [UDM.0,UDM.2]	RD[*FOM*_av_(A,B)] [UDM.2,UDM.3]	RD[*FOM*_av_(A,B)] [UDM.3,UDM.3y]	RD[*FOM*_av_(A,B)] [AEM,UDM.3]
2.55	2.58	7.69	0.44	0.04	4.85

## Data Availability

The original contributions presented in this study are included in the article. Further inquiries can be sent to the corresponding author.

## Abbreviations

The following abbreviations are used in this manuscript:

UV–Vis–NIR	Ultraviolet–visible–near infrared
a-Si	Amorphous silicon
MS	Magnetron sputtering
TLU	Tauc–Lorentz–Urbach dispersion model
TLUF TLU	Dispersion model of Foldyna et al.
TLUR TLU	Dispersion model of Rodriguez et al.
GO	Gaussian oscillator
UDM	Universal dispersion model
UDM.0	UDM without Gaussian oscillators
UDM.2	UDM with two Gaussian oscillators
UDM.3	UDM with three Gaussian oscillators
AEM	Advanced envelope method
a-Si:H	Amorphous silicon containing 5–15% hydrogen
JDOS	Joint density of states
CEEMDAN	Complete ensemble empirical mode decomposition with adaptive noise
SMEDM	Smoothed median envelope denoising method
FOM	Figure of merit
DOS	Density of states
D0	Neutral dangling bond
D+	Positive dangling bond
D−	Negative dangling bond
PECVD	Plasma-enhanced chemical vapor deposition
